# 5-Hydroxymethylfurfural Mitigates Lipopolysaccharide-Stimulated Inflammation via Suppression of MAPK, NF-κB and mTOR Activation in RAW 264.7 Cells

**DOI:** 10.3390/molecules24020275

**Published:** 2019-01-13

**Authors:** Fanhui Kong, Bae Hoon Lee, Kun Wei

**Affiliations:** 1 School of Bioscience and Bioengineering, South China University of Technology, Guangzhou 510006, Guangdong, China; kong_fanhui@foxmail.com; 2 Wenzhou Institute of Biomaterials and Engineering, CAS, Wenzhou 325011, Zhejiang, China; 3 School of Ophthalmology & Optometry, School of Biomedical Engineering, Wenzhou Medical University, Wenzhou 325027, Zhejiang, China

**Keywords:** 5-hydroxymethylfurfural, RAW 264.7, inflammation, inflammatory cytokines, MAPK, Akt/mTOR, NF-κB

## Abstract

5-Hydroxymethylfurfural (5-HMF) is found in many food products including honey, dried fruits, coffee and black garlic extracts. Here, we investigated the anti-inflammatory activity of 5-HMF and its underlying mechanisms in lipopolysaccharide (LPS)-stimulated RAW 264.7 cells. 5-HMF pretreatment ranging from 31.5 to 126.0 μg/mL reduced the production of nitric oxide (NO), prostaglandin E2 (PGE2) and pro-inflammatory cytokines (TNF-α, IL-1β and IL-6) in a concentration-dependent manner in LPS-stimulated cells. Moreover, 5-HMF-pretreated cells significantly down-regulated the mRNA expression of two major inflammatory mediators, nitric oxide synthase (iNOS) and cyclooxygenase-2 (COX-2) and suppressed the production of pro-inflammatory cytokines, as compared with the only LPS-stimulated cells. 5-HMF suppressed the phosphorylation of extracellular regulated protein kinases (ERK1/2), c-Jun N-terminal kinase (JNK), IκBα, NF-κB p65, the mammalian target of rapamycin (mTOR) and protein kinase B (Akt). Besides, 5-HMF was proved to inhibit NF-κB p65 translocation into nucleus to activate inflammatory gene transcription. These results suggest that 5-HMF could exert the anti-inflammatory activity in the LPS-induced inflammatory response by inhibiting the MAPK, NF-κB and Akt/mTOR pathways. Thus, 5-HMF could be considered as a therapeutic ingredient in functional foods.

## 1. Introduction

Inflammation, as a normal, self-protective and highly regulated mechanism of the human body, is a complex biological response to external and internal stimuli such as infection and tissue injury. It can also prevent attacks caused by pathogenic microorganisms, heal tissue damage and restore normal tissue structure [1,2]. In general, inflammation is beneficial and useful for physiological functions of the body; however, it can lead to tissue damage or multiple chronic diseases such as cancer, diabetes, cardiovascular diseases, rheumatoid arthritis and neurological disorders when inflammation is prolonged or excessive [3,4,5]. Especially, chronic low-grade inflammation (silent inflammation) has been thought to lead to development of many diseases of later life (arthritis, heart disease and cancer) [6]. Also, low-grade inflammation is considered to be associated with inflammatory diets, toxic metals, alcohol or stress [7]. Pharmaceutical medicine to suppress and remedy inflammatory diseases has revealed prominent effectiveness and has been widely prescribed in metabolic diseases such as type 2 diabetes and cardiovascular diseases [8]. However, the long-term use of non-steroidal anti-inflammatory drugs (e.g., Aspirin and Ibuprofen) is often related to the adverse side effect [9]. The daily intake of natural anti-inflammation molecules to suppress chronic inflammation and alleviate inflammatory-associated symptoms could be a permanent and valid strategy. Recently, anti-inflammatory health foods/supplements such as omega-3 fatty acids, antioxidants and vitamins have drawn growing attention because they might prevent or reduce silent inflammation [10,11].

5-Hydroxymethylfurfural (5-HMF) is present in various food products including honey, dried fruits, coffee and fruit juices [12]. 5-HMF, a product of the common heterocyclic Maillard reaction, is mainly produced by acid-catalyzed thermal dehydration of fructose and used as a flavoring substance in heat-processed products [13]. In addition, 5-HMF has been found in a great deal of heat-processed Chinese traditional medicine such as black garlic extracts [14]. Some studies have reported that aged black garlic exhibited anti-inflammatory, antioxidant and anti-cancer properties [15,16,17]. Moreover, other research reports showed that black garlic was effective in alleviating the inflammation via the inhibition of nitric oxide (NO) production [16,17]. However, 5-HMF has been subjected to widespread controversy regarding its toxicity and carcinogenicity [18,19]. Recently, it has been reported that 5-HMF featured many positive biological functions such as antioxidant activity and protection against acute hypobaric hypoxia [20,21,22]. Moreover, 5-HMF was conducive to inhibition of alcoholic liver oxidative injury [23] and also exerted cardioprotective effects [24]. As far as we know, the direct effect of 5-HMF on anti-inflammatory activity has not been scrutinized systematically. In this report, we assessed the anti-inflammatory potency of 5-HMF in lipopolysaccharide-stimulated RAW 264.7 cells and explored its underlying anti-inflammatory mechanisms.

## 2. Results

### 2.1. Cell Viability of 5-HMF

**Figure 1:**
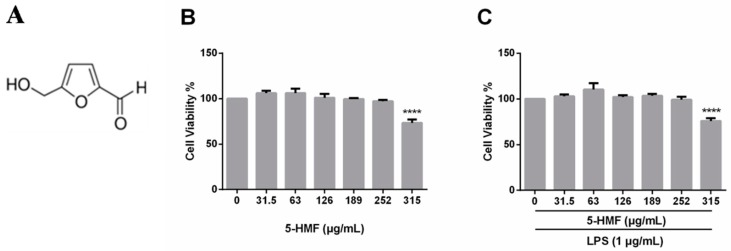
Cell viability of 5-HMF. (**A**) The chemical structure of 5-hydroxymethylfurfural. (**B**) Effects of 5-HMF on the cell viability in RAW 264.7 cells. Cells were treated with various concentrations of 5-HMF for 24 h, followed by the MTT assay. (**C**) Effects of 5-HMF in the presence of LPS on the cell viability in LPS-induced RAW 264.7 cells. Cells were treated with various concentrations of 5-HMF for 6 h and then further treated with LPS (1 μg/mL) for 24 h. The data are expressed as means ± SDs (n = 3). ****: P < 0.001, as compared with the blank control group.

The cytotoxicity of 5-HMF on RAW 264.7 cells was tested in the absence or presence of LPS (1 μg/mL) by the MTT assay. First, cells were pretreated with only 5-HMF for 24 h for assessment of the cytotoxicity of pure 5-HMF. In another experiment, cells were pretreated with 5-HMF for 6 h, followed by LPS treatment for 24 h. 5-HMF exhibited little cytotoxicity up to a concentration of 252.0 μg/mL (Figure 1B). Furthermore, even the presence of LPS (1 μg/mL) did not affect the cell viability under the current culture conditions, either. Therefore, 5-HMF at concentrations of 0, 31.5, 63.0 and 126.0 μg/mL was used in the subsequent experiments.

### 2.2. The Effect of 5-HMF on NO and PGE2 Production

**Figure 2:**
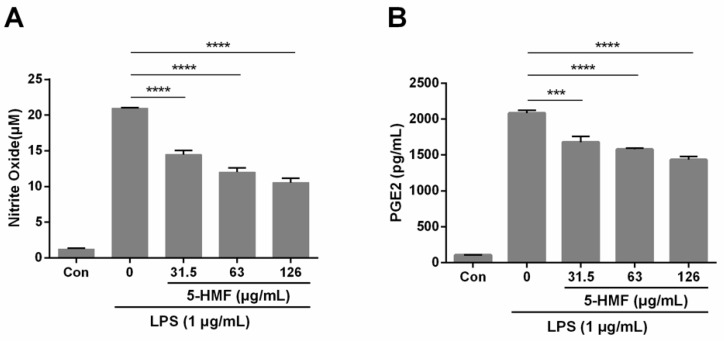
The suppressive effect of 5-HMF on Nitric Oxide production (NO) and prostaglandin E2 (PGE2) in RAW 264.7 cells. Cells were pretreated with various concentrations of 5-HMF for 6 h prior to the stimulation of LPS (1 μg/mL) for 18 h. (**A**) The NO levels in the cell media were measured by Griess assays. NO production in the culture media decreased with increasing the amount of 5-HMF pretreatment. (**B**) The PGE2 levels were measured using the ELISA kit. The PGE2 levels decreased with an increase in the amount of 5-HMF pretreatment. The results are expressed as means ± SDs (n = 3). ***: P < 0.005, ****: P < 0.001, compared with the only LPS-stimulated control group.

To evaluate the effect of 5-HMF on LPS-induced NO and PGE2 production, Griess reagent and ELISA kits were employed. When 1 μg/mL of LPS was introduced, the production of NO increased markedly compared with no LPS, which signifies that the inflammatory cellular model was effective. When RAW 264.7 cells were pretreated with 5-HMF, the NO (n = 3, one-way ANOVA, p < 0.001) and PGE2 (n = 3, one-way ANOVA, p < 0.005) production decreased significantly in a concentration-dependent manner, as compared with the LPS-stimulated control group. The percent inhibition of NO production in 31.5 μg/mL, 63.0 μg/mL and 126.0 μg/mL 5-HMF-treated groups was 30.90%, 42.71% and 49.70%, respectively (Figure 2).

### 2.3. The Effect of 5-HMF on the Expression of Pro-Inflammatory Cytokines (TNF-α, IL-1β and IL-6)

**Figure 3:**
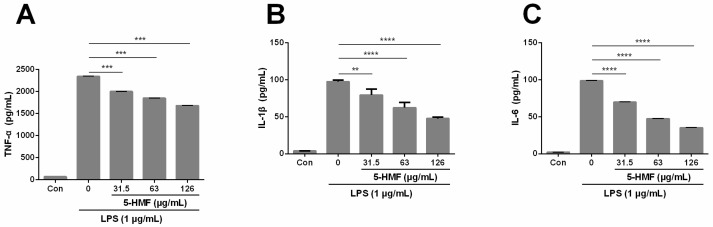
The effect of 5-HMF on the expression of TNF-α (**A**), IL-1β (**B**) and IL-6 (**C**) in LPS-activated RAW 264.7 cells. Cells were pretreated with various concentrations of 5-HMF for 6 h prior to the stimulation of LPS (1 μg/mL) for 18 h. The concentration of TNF-α, IL-6 and IL-1β in the culture medium was analyzed using the respective ELISA kits, respectively. Data were expressed as means ± SDs (n = 3). **: P < 0.01, ***: P < 0.005, ****: P < 0.001, compared with the only LPS-stimulated control group.

The effect of 5-HMF on the expression of pro-inflammatory cytokines (TNF-α, IL-1β and IL-6) in LPS-induced RAW 264.7 cells was assessed with the respective ELISA kits. The results showed that when cells were pretreated with 5-HMF, the expression levels of TNF-α (Figure 3A), IL-1β (Figure 3B) and IL-6 (Figure 3C) were significantly attenuated in a concentration-dependent manner, as compared with the group treated with LPS only (n = 3, one-way ANOVA, P < 0.0005). These results indicate that 5-HMF is capable of suppressing the production of pro-inflammatory cytokines such as TNF-α, IL-1β and IL-6.

### 2.4. The Effect of 5-HMF on LPS-Stimulated iNOS, COX-2, TNF-α, IL-1β and IL-6 mRNA Expression

**Figure 4:**
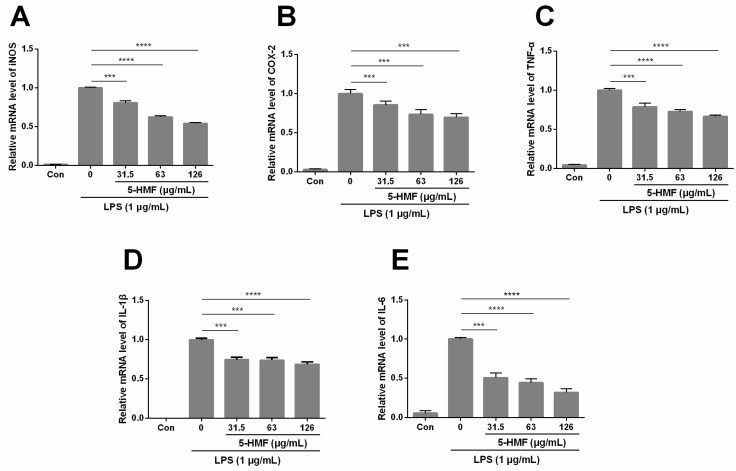
The effect of 5-HMF on the mRNA expression level of iNOS (**A**), COX-2 (**B**), TNF-α (**C**), IL-1β (**D**) and IL-6 (**E**) in LPS-activated RAW 264.7 cells. RAW 264.7 cells were pre-treated with 5-HMF (0, 31.5, 63.0 and 126.0 μg/mL) for 6 h prior to the stimulation for 18 h with LPS (1 μg/mL). The mRNA levels were analyzed by real-time RT-PCR. The results represent relative mRNA expression levels, which were normalized to the reference gene (β-actin). The relative IL-1β expression level of the control was 2.120 × 10^−5^ ± 0.366 ×10^−5^. Data were expressed as means ± SDs (n = 3). *: P < 0.05, **: P < 0.01, ***: P < 0.005, ****: P < 0.001, compared with the LPS-stimulated group.

To elucidate the role of 5-HMF in the anti-inflammatory effect in LPS-stimulated RAW 264.7 cells at the molecular level, the mRNA expression level of two main inflammatory mediators (iNOS and COX-2) and pro-inflammatory cytokines (TNF-α, IL-1β and IL-6) was investigated through the qPCR analysis. As presented in the Figure 4A,B, 5-HMF effectively inhibited the expression of iNOS (n = 3, one-way ANOVA, P < 0.0001) and COX-2 (n = 3, one-way ANOVA, P < 0.05) in a concentration-dependent manner, as compared with the only LPS-induced control group. As the concentration of 5-HMF rose, the expression levels of iNOS and COX-2 were suppressed ranging from 20–48% and 15–30%, respectively. Also, the expression levels of TNF-α and IL-6 cytokines were remarkably decreased compared with the only LPS-induced control group (P < 0.05). 

### 2.5. The Effect of 5-HMF on the ROS Content

**Figure 5:**
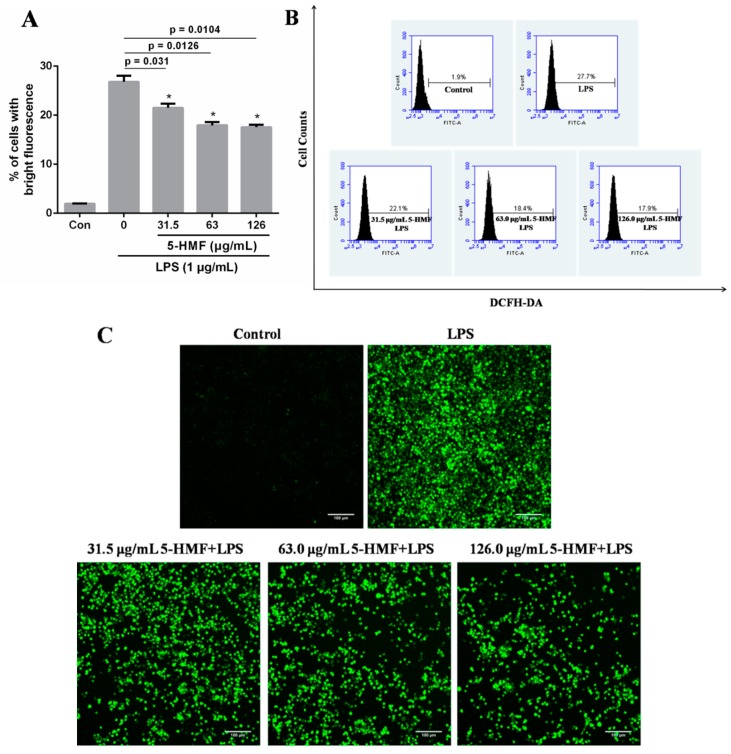
The effect of 5-HMF on the ROS content. The quantitative analysis of intracellular ROS formation in LPS-activated RAW 264.7 cells using flow cytometry. (**A**) The ROS content was expressed as the percentage of cells with bright fluorescence. (**B**) The flow cytometry chart was analyzed by the software (BD Accuri C6 Plus). (**C**) Confocal microscopy images of cells with intracellular ROS. The green fluorescence represents ROS. 5-HMF inhibited LPS-stimulated ROS production in RAW 264.7 cells. RAW 264.7 macrophage cells were pretreated for 6 h with different concentrations of 5-HMF prior to the stimulation of LPS (1 μg/mL) for 18 h. Data were expressed as means ± SDs (n = 3). *: P < 0.05, compared with the only LPS-induced control group. Scale bar = 100 μm.

Inflammatory stimulation can induce aggregation and activation of immune cells such as macrophages and neutrophils and release a large amount of reactive oxygen and nitrogen species. As shown in Figure 5A, after RAW 264.7 cells were treated with LPS, the ROS content markedly escalated, as compared with the blank control group. However, when cells were pretreated with 5-HMF, the ROS content decreased by around 10%, which manifested that 5-HMF could suppress the ROS content in the inflammatory reaction. In addition, the intracellular ROS image results were consistent with the flow cytometry results, as displayed in Figure 5B,C. Therefore, 5-HMF can inhibit the ROS production in LPS-stimulated RAW 264.7 cells.

### 2.6. The Effect of 5-HMF on the MAPK, Akt/mTOR and NF-κB Signaling Pathways

**Figure 6:**
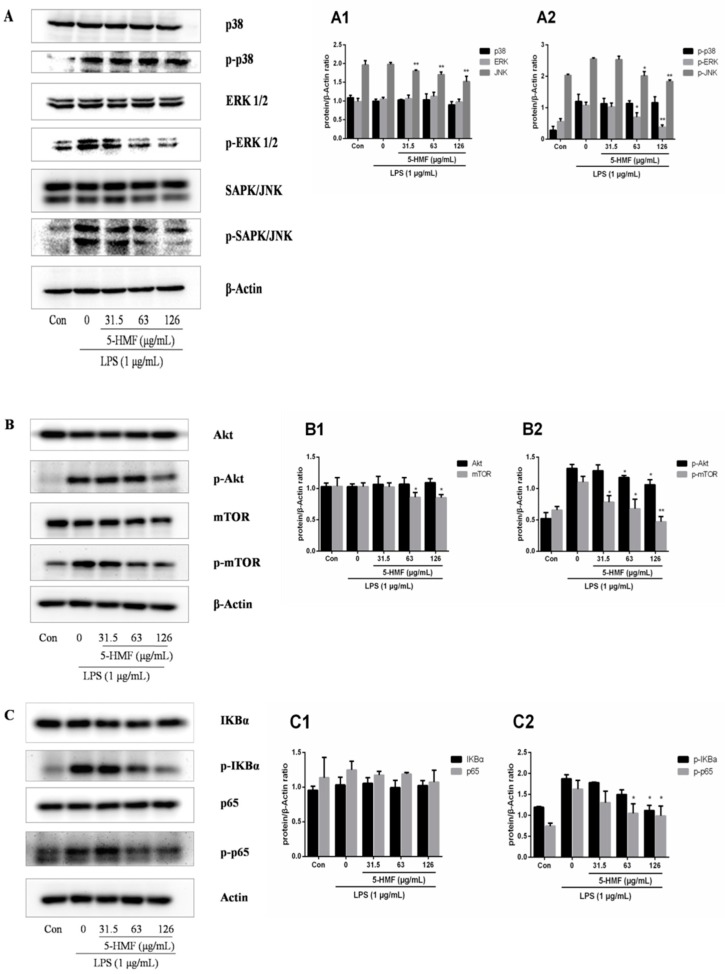
The effect of 5-HMF on the protein expression levels of the MAPK (**A**), Akt/mTOR (**B**) and NF-κB (**C**) in LPS-activated RAW 264.7 cells. A1, A2, B1, B2, C1 and C2 show the gray value analysis results of Western blot bands on the protein expression levels of related signal pathways. RAW 264.7 cells were pretreated with 5-HMF (0, 31.5, 63.0 and 126.0 μg/mL) for 6 h and then stimulated using LPS (1 μg/mL) for 18 h. β-Actin served as the internal reference. *: P < 0.05, **: P < 0.01, compared with the LPS-stimulated control group.

To gather some insight into molecular mechanisms involved in the suppressive effect of 5-HMF on LPS-stimulated inflammation in RAW 264.7 cells, western blot assays on the MAPK, Akt/mTOR and NF-κB signal pathways were conducted. The MAPK pathways, including p38, extracellular regulated protein kinases (ERK1/2) and c-Jun N-terminal kinase (c-JNK) regulate the expression of pro-inflammatory genes in response to external stimuli. The results showed that the expression levels of p38, ERK1/2, Akt, mTOR, IκBα and p65 were similar among the groups (the blank control, the LPS-stimulated control and 5-HMF-pretreated groups). In addition, phosphorylated proteins (p-p38, p-ERK1/2, p-SAPK/JNK, p-Akt, p-mTOR, p-IκBα and p-p65) were marginally expressed in the blank control group whereas those in the LPS-stimulated group were highly expressed. However, the expression levels of p-ERK1/2, p-JNK, p-Akt, p-mTOR, p-IκBα and p-p65 were markedly reduced in the 5-HMF-pretreated group, compared with the only LPS-activated control group whereas the expression of p-p38 upregulated by the activation of LPS was not suppressed significantly (Figure 6A).

### 2.7. The Effect of 5-HMF on the NF-κB p65 Nuclear Translocation

**Figure 7:**
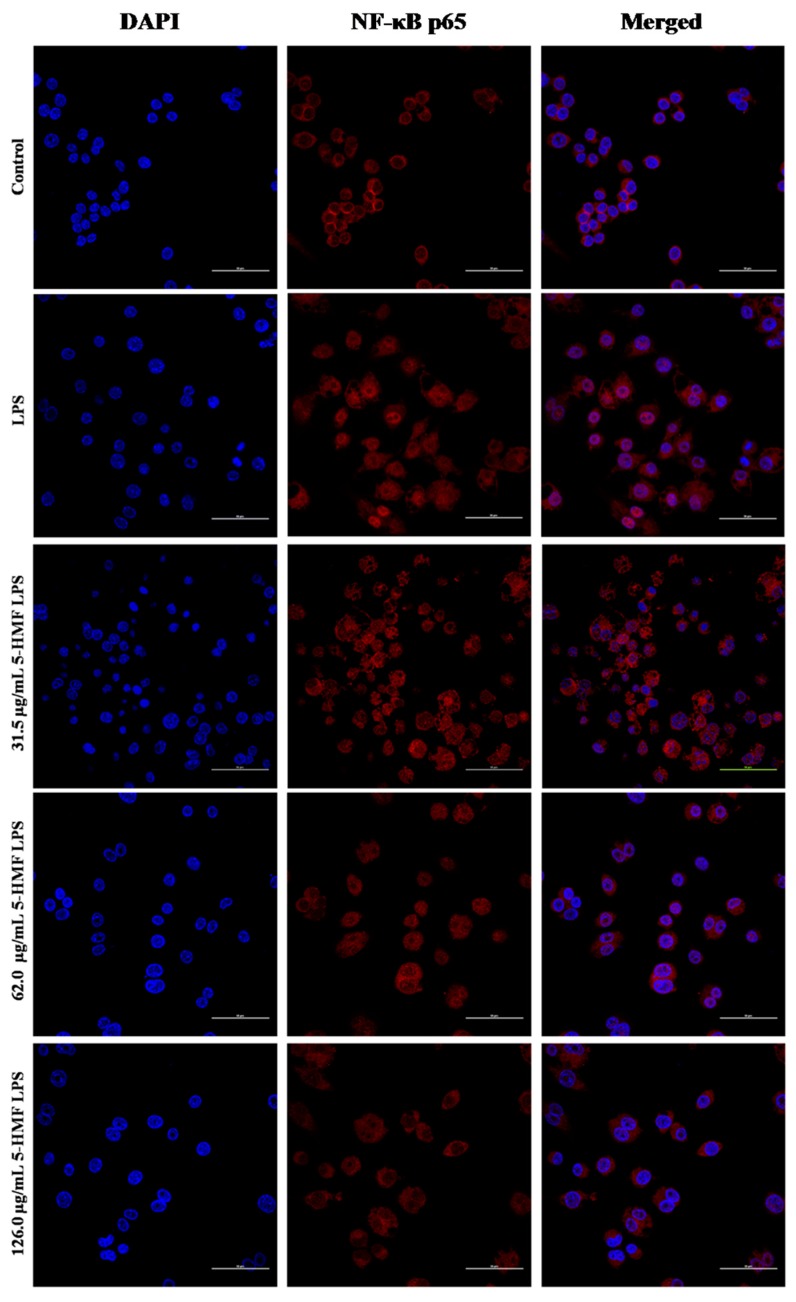
5-HMF inhibited LPS-stimulated NF-κB p65 nuclear translocation in RAW 264.7cells. Scale bar = 50 μm. Nuclear translocation of NF-κB p65 was assessed using laser scanning confocal microscopy. RAW 264.7 cells were pretreated with different concentrations of 5-HMF for 6 h before the activation of LPS (1 μg/mL) for 18 h. The cells were stained with NF-κB p65 antibodies (red) and DAPI (blue).

The inhibition effect of 5-HMF on the NF-κB p65 activation was evaluated with a laser scanning confocal microscope. As shown in Figure 7, the unprovoked RAW 264.7 cells of NF-κB p65 (red color) were mainly distributed in the cytoplasm, whereas NF-κB p65 mostly emerged in the nucleus after LPS stimulation, which was reversed by 5-HMF pretreatment with varying the concentration of 5-HMF from 31.5 to 126.0 μg/mL. Taken together with the western blot analysis, these results suggest that 5-HMF could inhibit NF-κB p65 translocation into nucleus through suppressing the phosphorylation of p65 and IκBα.

## 3. Discussion

Dietary components are thought to be linked to low-grade inflammation, which may result in chronic diseases of later life such as cancer, cardiovascular diseases and Alzheimer’s disease [6,25]. Therefore, exploring a dietary compound with anti-inflammatory activity to ameliorate or cure inflammation has widespread appeal. It is well accepted that the natural food ingredients have a great deal of advantageous biological functions.

5-HMF is present in daily diets including breakfast cereal, coffee, honey and baked foods [12]. 5-HMF was known to exhibit healthy beneficial effects due to its anti-sickling [26,27], anti-allergic [28] and anti-oxidative properties [12,23]. Besides, it has been reported that the Maillard reaction products containing 5-HMF exerted antioxidant and anti-inflammatory activities in interferon γ- and phorbol ester-induced Caco-2 cells [29]. People normally take in 30–150 mg of 5-HMF daily through various food products [12]. However, the anti-inflammatory mechanisms of 5-HMF have not been fully elucidated. In this study, to explore the anti-inflammatory molecular mechanisms of 5-HMF, the mRNA and protein expression of inflammatory factors and their relevant signal pathways were investigated in LPS-stimulated RAW 264.7 cells. For cell viability of 5-HMF, 5-HMF was nontoxic to RAW 264.7 cells at concentrations of below 256.0 μg/mL. Thus, 5-HMF with different concentrations (0, 31.5, 63.0 and 126.0 μg/mL) was used to evaluate its effect on anti-inflammatory activities in LPS-induced RAW 264.7 cells. 

Macrophages are prominent innate immune effector cells with many functions and roles and act as sensors and responders to inflammation and immune regulations [30,31]. Inflammation, as a biological and physiological response to pathogens, injury as well as other stimuli, is essential for the body function and protecting from varieties of infection. Generally, macrophages participate in and promote immune responses of inducing the expression of pro-inflammatory cytokines (TNF-α, IL-6 and IL-1β) when stimulated by the exogenous substances (e.g., LPS). Meanwhile, the MAPK and NF-κB signaling pathways are activated through intracellular signaling cascades to produce inflammatory cytokines and regulate the LPS-induced macrophages inflammatory response [32]. In this study, macrophage-like RAW 264.7 cells were successfully activated by LPS treatment (1 μg/mL) for 18 h. LPS-activated RAW 264.7 cells exhibited immune responses such as the increased production of pro-inflammatory cytokines (e.g., TNF-α, IL-1β and IL-6) and the up-regulated expression of inflammatory mediators (e.g., iNOS and COX-2). 

TNF-α takes part in a number of cellular processes, including cell survival, apoptosis and necrosis. TNF-α plays a crucial role in regulating immune responses and inflammation processes through activation of TNF receptors and relevant signal pathways such as NF-κB or MAPKs [33,34]. Normally, IL-6 is strictly regulated by transcriptional and posttranscriptional mechanisms and is implicated in inflammation and autoimmunity. However, the dysregulated expression of IL-6 can lead to development of chronic inflammation or abnormal autoimmunity [35]. IL-1β is a member of the IL-1 family and acts as a crucial mediator of the inflammatory response [36]. In this study, when macrophage-like RAW 264.7 cells were pretreated with 5-HMF, the production of pro-inflammatory cytokines (e.g., TNF-α, IL-6 and IL-1β), both in mRNA and protein expression levels, were suppressed in comparison with the only LPS-induced control group (P < 0.01). These results suggest that 5-HMF can exhibit a protective and repressive role in LPS-stimulated RAW 264.7 macrophage cells.

NO and PGE2 are the crucial signal transduction molecules of inflammatory responses. As a bioactive lipid, PGE2 has many physiological functions in connection with inflammation. Moreover, PG is synthesized through COX, which has two isoenzymes (COX-1 and COX-2). COX-2 is closely related to inflammation because PGE2 is primarily produced via mediation of COX-2 in LPS-induced macrophages [37]. Therefore, inhibiting COX-2 expression is one of the effective ways to suppress PGE2 production and to alleviate symptoms of inflammation. On the other hand, NO is synthesized and released through a series of reactions by nitric oxide synthases, which include neuronal NOS (nNOS), endothelial NOS (eNOS) and inducible NOS (iNOS) [38]. NO is mainly regulated by iNOS. Increased iNOS expression can result in many disorders of chronic inflammation [39]; therefore, it is important to restrain NO or iNOS over-expression for anti-inflammation. 5-HMF significantly suppressed NO production by down-regulating iNOS mRNA expression in a dose-dependent manner. Moreover, the PGE2 and COX-2 expression levels were lowered in the 5-HMF-pretreated groups, as compared with the LPS-induced control group, which indicates that 5-HMF also has the ability to inhibit the inflammatory mediator production.

Reactive oxygen species (ROS) are involved in inflammatory disorders and immune regulation [40], which are central to the progression of a great number of inflammatory diseases [41]. Enhanced and prolonged ROS generation can cause endothelial dysfunction, tissue injury as well as many chronic diseases such as inflammatory bowel disease [40]. Therefore, it is necessary to regulate and control the ROS generation to prevent from aggravation of inflammation. The pretreatment with 5-HMF attenuated the ROS production relative to the LPS-stimulated control group. 5-HMF has several functional electron-active groups (one aldehyde, one hydroxyl group and two double bonds and one oxygen in its furan structure), which could interact with ROS and potentially scavenge them [12,42].

TLR4, one of the Toll-like receptor (TLR) family members, is also a primary receptor in the LPS-stimulated reaction [43] and specifically recognizes LPS [2,44]. It is well known that the inflammatory reaction of LPS-stimulated macrophages conveys multiple intracellular and extracellular protein interactions [45]. When the TLR4 extracellular domains are stimulated by LPS, the response is triggered through primarily related signaling pathways including MAPKs, Akt/mTOR and the NF-κB [46]. NF-κB and MAPKs are vital intracellular signaling pathways which are stimulated by LPS and in turn activate the expression of multiple inflammatory cytokines and mediators such as COX-2 and iNOS [47]. It is considered that targeting TLR4 is safe to alleviate chronic inflammatory conditions without injuring the innate immune response [48]. MAPKs mainly include ERK1/2, SAPK/JNK and p38 protein kinases, which are involved in many physiological processes such as cellular proliferation, apoptosis, differentiation and inflammatory responses [49]. In the LPS-stimulated macrophage cells, the MAPK proteins are activated to act on their substrates to regulate the inflammatory reaction. One report demonstrated that suppression of the p38 pathway distinctly decreased the protein expression of iNOS and COX-2, thus further mitigating the inflammatory response [50]. Besides, the pro-inflammatory cytokines were found to stimulate the three isoforms of MAPKs. Therefore, the MAPK signal pathway is significant in the regulation of the inflammatory responses. In this study, 5-HMF exerted its anti-inflammation effect through down-regulating iNOS and COX-2 mRNA and protein expression levels and inhibiting the phosphorylation of JNK and ERK in the MAPK signal pathway in macrophage cells. The results indicated that the 5-HMF can mitigate the inflammatory reaction through suppressing the MAPK signal pathway. 

Besides, it was reported that activation of the MAPK signaling pathway by LPS could indirectly activate the downstream NF-κB pathway and stimulate complex physiological responses [51]. NF-κB is a vital downstream signal pathway in the LPS-stimulated reaction, which is associated with tumor growth and inflammation [52]. The transcription factor NF-κB is considered as the paramount regulator of the inflammatory response; the inflammation can lead to degradation of the inhibitory protein of NF-κB and dissociation of NF-κB from its inhibitory complex when cells are stimulated by LPS. Moreover, NF-κB can initiate the transcription of hundreds of genes including enzymes and cytokines while the uncombined NF-κB translocates to the cell nucleus [53,54]. The inactive IκBα/p65 form bound to IκBα in the cytoplasm is degraded, while the active p65 is translocated into the nucleus to bind to pro-inflammatory genes, which can trigger the inflammatory response in LPS-induced macrophages [55]. It was reported that Sulfasalazine, an anti-inflammatory agent, exerted the anti-inflammation effect through the inhibition of the NF-κB pathway, suppression of IκBα phosphorylation and its subsequent degradation [56]. Besides, one of the members of the NF-κB family (p50) could regulate the pro- and anti-inflammatory cytokine production, which may provide potential new methods to adjust and control the innate immune response [57]. However, as p50 lacks a transactivation domain, p50 is thought as a regulatory subunit to modulate the DNF binding affinity of p65. Therefore, the protein expression of IκBα and the translocation of p65 are considered as the crucial indicators of the inflammation [58]. In this current study, 5-HMF exerted the anti-inflammatory and protective effect through down-regulating the protein phosphorylation of IκBα and p65 and subsequently inhibiting the p65 nuclear translocation in the LPS-stimulated RAW 264.7 cells. 

The phosphatidylinositol 3-kinases/Akt/mammalian target of the rapamycin (PI3K/Akt/mTOR) signaling pathway, has involved in autophagy-regulatory and inflammation [59]. Also, PI3K/Akt/mTOR has multiple aspects in inflammatory regulation [60]. It was reported that the PI3K/Akt/ mTOR pathway served as a leader to control the cellular response to pathogens through limiting the pro-inflammatory responses in myeloid phagocyte [61]. In this study, 5-HMF lowered the protein phosphorylation of Akt and mTOR in LPS-induced RAW 264.7 cells, implying that 5-HMF might be involved in the Akt and mTOR pathway.

**Figure 8:**
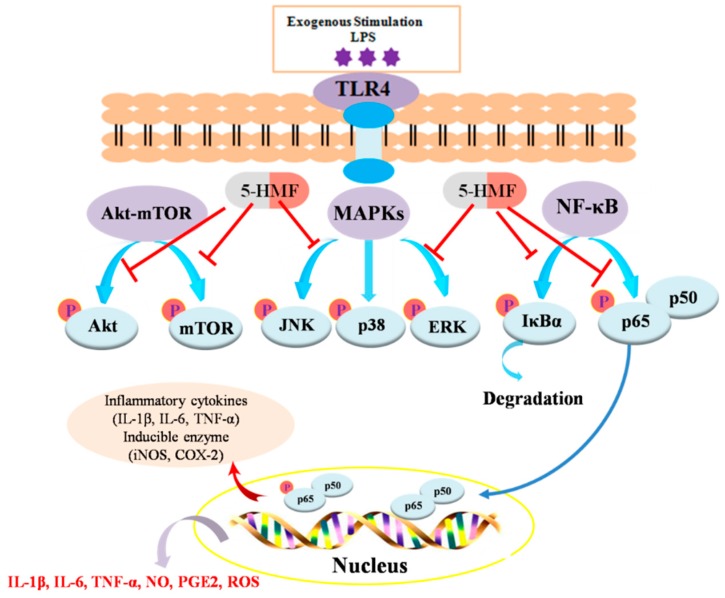
Suggested anti-inflammatory mechanisms of 5-HMF in LPS-stimulated macrophages.

Taken together, 5-HMF was found to inhibit the production of inflammatory cytokines (NO, PGE2, TNF-α, IL-6 and IL-1β) and ROS. These results support that 5-HMF could mitigate the inflammatory response in LPS-stimulated RAW 264.7 macrophage via suppressing the protein phosphorylation of MAPK, NF-κB and Akt/mTOR signal pathways, as was illustrated in Figure 8. Hence, 5-HMF might be considered as a potential therapeutic component in food products.

## 4. Materials and Methods 

### 4.1. Chemicals and Reagents

LPS (*Escherichia coli* O111:B4) and 5-HMF were purchased from Sigma-Aldrich (St. Louis, MO, USA). Dulbecco’s Modified Eagle Medium (DMEM) was purchased from Gibco (ThermoFisher, Gaisburg, MD, USA). Fetal bovine serum (FBS) was purchased from Lonsera (Lonsa Science SRL, Guichon, Uruguay). MTT cell proliferation and cytotoxicity assay kit was purchased from Phygene life sciences (Fuzhou, China). The primary rabbit monoclonal antibodies against p38 MAPK (D13E1) XP^®^, SAPK/JNK Antibody, p44/42 MAPK (ERK1/2) (137F5), phospho-p38 MAPK (Thr180/Tyr182) (D3F9) XP^®^, phospho-SAPK/JNK (Thr183/Tyr185) (81E11), phospho-p44/42 MAPK (ERK1/2) (Thr202/Tyr204) (D13.14.4E) XP^®^, Akt (pan) (C67E7), mTOR (7C10), phospho-Akt(Ser473) (D9E) XP^®^, phospho-mTOR (Ser2448) (D9C2) XP^®^, IκBα (44D4), NF-κB p65 (D14E12) XP^®^, phospho-IκBα (Ser32) (14D4) and phospho-NF-κB p65 (Ser536) were purchased from Cell Signaling Technology (Danvers, MA, USA).

### 4.2. Cell Culture

Murine macrophage-like cell line RAW 264.7 was purchased from American Type Cell Culture (ATCC, Rockefeller, MD, USA). RAW 264.7 cells were cultured in DMEM supplemented with 10% FBS and 1% Penicillin-Streptomycin (100 U/mL penicillin and 100 mg/mL streptomycin) in a humidified incubator at 37 °C with 5% CO_2_ atmosphere.

### 4.3. Cell Viability Assay

Cell viability was tested by the colorimetric MTT reagent assay. RAW 264.7 cells were seeded (5 × 10^3^ cells/each well) in a 96-well plate and cultured overnight. After pretreatment with different concentrations (0, 31.5, 63.0, 126.0, 189.0, 252.0 and 504.0 μg/mL) of 5-HMF for 6 h, the cells were stimulated with or without 1 μg/mL of LPS for 24 h. After removal of the media, 110 μL of the MTT solution (10 μL MTT plus 100 μL culture medium) was added to each well and incubated for 4 h. Then the media were removed and 100 μL of the formazan solvent was added to dissolve the purple precipitates. The absorbance was measured at a wavelength of 570 nm using a Modular multitechnology microplate reader (Thermo Fisher Scientific, Gaisburg, MD, USA). Each sample group consisted of at least three replicates.

### 4.4. Nitric Oxide (NO) Measurement

The amount of NO in the culture medium was determined by Griess reagent (Beyotime, Shanghai, China). RAW 264.7 cells were seeded in a 12-well plate (3 × 10^5^ cells/each well) and incubated at 37 °C with 5% CO_2_ for 24 h. After that, the cells were pretreated with 5-HMF at various concentrations (0, 31.5, 63.0 and 126.0 μg/mL) for 6 h and afterward stimulated with LPS (1 μg/mL) for 18 h. Then, 50 μL of a supernatant medium from each well was transferred into a 96-well plate and subsequently, Griess I reagent (50 μL) and Griess II reagent (50 μL) were added to each well. The absorbance was measured at a wavelength of 540 nm using the Modular multitechnology microplate reader. Two controls (a blank control and a normal control) were prepared as follows: The blank control was a sample group without 5-HMF pretreatment and LPS stimulation; the normal control was a sample group without 5-HMF pretreatment but with LPS stimulation.

### 4.5. Measurement of Cytokine (TNF-α, IL-1β and IL-6) and PGE2 Levels

RAW 264.7 cells were seeded in a 12-well plate (3 × 10^5^ cells/each well) and incubated at 37 °C with 5% CO_2_ for 24 h. After that, the cells were pretreated with 5-HMF at various concentrations (0, 31.5, 63.0 and 126.0 μg/mL) for 6 h and then stimulated with LPS (1 μg/mL) for 18 h. Each medium was collected in a tube and centrifuged at 1000 rpm for 10 min. The expression levels of TNF-α, IL-1β and IL-6 were determined with the mouse ELISA kits (Boster Biological Technology, Wuhan, China) and PGE2 was determined with the mouse ELISA kits (CUSABIO, Shanghai, China) as per each manufacturer’s instructions.

### 4.6. Quantitative Reverse Transcriptase-Polymerase Chain Reaction (qRT-PCR)

RAW 264.7 cells were seeded in a 12-well plate (3 × 10^5^ cells/each well) and incubated at 37 °C with 5% CO_2_ for 24 h. and stimulated with LPS (1 μg/mL) for 18 h after a 6-h pre-treatment with 5-HMF at different concentrations (0, 31.5, 63.0 and 126.0 μg/mL). Total cellular RNA was isolated from cultured RAW 264.7 cells using Trizol reagent (Sangon Biotech, Shanghai, China). The RNA was reverse-transcribed into cDNA using ReverTra Ace^®^ qPCR RT Master Mix with gDNA Remover (TOYOBO LIFE SCIENCE, Shanghai, China) in T100™ Thermal Cycler (BIO RAD, Hercules, CA, USA). The sample mixture was prepared with 1.5 μL of cDNA in 10 μL of ChamQ SYBR qPCR Master Mix (Vazyme Biotech, Nanjing, China), primers and ddH_2_O for a final volume of 20 μL. The primers were supplied from Sangon Biotech (Shanghai, China) and the sequences are listed in Table 1. The qPCR was carried out using the LightCycler^®^ 96 Real-Time PCR System (Roche, Switzerland) under the standard thermal cycle conditions: 95 °C for 30 s, 40 cycles of 95 °C for 10 s and 60 °C for 30 s, followed by 95 °C for 10 s, 65 °C for 60 s and 97 °C for 1 s. The threshold cycle (CT) was calculated as the fractional cycle number at which the amount of the amplified target gene reached a fixed threshold. β-actin was used as the housekeeping gene. Each reaction in at least three independent experiments was performed in triplicate.

### 4.7. Reactive Oxygen Species (ROS) Measurement

RAW 264.7 cells were seeded in a 12-well plate (3 × 10^5^ cells/each well) and incubated at 37 °C with 5% CO_2_ for 24 h. After that, the cells were pretreated with 5-HMF with various concentrations (0, 31.5, 63.0 and 126.0 μg/mL) for 6 h and subsequently stimulated with LPS (1 μg/mL) for 18 h. Later, the cells were washed using cold PBS three times and stained by 10 μM 2,7-Dichlorodi-hydrofluorescein diacetate (DCFH-DA) dissolved in serum-free media for 30 min. The DCFH-DA fluorescence morphology was examined using a laser scanning confocal microscope (Zeiss, LSM 710, German). For flow cytometry, cells were also detached via trypsin after removal of the medium and PBS washing. After centrifugation, PBS was used for re-suspension of cell pellets and ROS of the cells were detected through a flow cytometer (Beckman Coulter CytoFLEX, Boulevard Brea, CA, USA). The results of the flow cytometry test were analyzed using the BD Accuri C6 Plus software (Becton Dickinson, Franklin, NJ, USA).

### 4.8. Western Blot Analysis

RAW 264.7 cells (1 × 10^6^ cells/each well in 6-well plates) were pre-treated with 0, 31.5, 63.0 and 126.0 μg/mL of 5-HMF for 6 h and then stimulated by 1 μg/mL of LPS for 18 h. The cells were washed with cold PBS and then total protein extraction was conducted with the RIPA lysis buffer (Beyotime, China) on ice. The protein concentration was assessed with a BCA protein assay kit (Sangon Biotech, Shanghai, China). Western blot analysis was carried out using the standard protocol: the quantified proteins (15 μg) were subjected to electrophoresis on 12% SDS-PAGE gels for 90 min and then transferred to PVDF membranes for 2 h (Mini Gel Tank, Invitrogen, Gaisburg, MD, USA). The PVDF membranes were blocked in 5 % skimmed-milk in TBST for 2 h and washed with TBST three times and then they were incubated with primary rabbit monoclonal antibodies against p38, JNK, ERK1/2, p-p38, p-JNK, p-ERK1/2, Akt, p-Akt, mTOR, p-mTOR, IκBα, p65, p-IκBα, p-p65 and β-Actin at 4 °C overnight. Next, they were washed three times with TBST and incubated with an anti-rabbit HRP-linked antibody (Cell Signaling Technology, Danvers, MA, USA) at a 1:8000 dilution at room temperature for 1 h. The proteins were visualized using Immobilon Western Chemiluminescent HRP Substrate (Millipore Corporation, Billerica, MA, USA) with the ChemiDoc-It Imaging System (UVP, Upland, CA, USA).

### 4.9. Immunofluorescence Staining

NF-κB nuclear translocation was examined by immunofluorescence microscopy. RAW 264.7 cells (5 × 10^4^ cells/each well in a 24-well plate with sterile coverslips) were pre-treated with 0, 31.5, 63.0 and 126.0 μg/mL of 5-HMF for 6 h and then stimulated by 1 μg/mL of LPS for 18 h. Cells were fixed with 4% paraformaldehyde for 15 min at room temperature and then washed with PBS twice. Later, the cells were washed with a quenching solution (0.1% glycine in PBS) twice, permeated with 0.1% Triton X-100 for 10 min and blocked using a blocking solution (10% FBS in PBS) for 1 h at room temperature. Then the cells were incubated with the primary p65 antibody at 4 °C overnight and later incubated with Conjugated Goat anti-Rabbit IgG H&L (Alexa Fluor^®^ 594) (1:2000, ab150080, Abcam, UK) for 1 h at room temperature and then stained with 4,6-diamidino-2 phenylindole (DAPI) for 5 min. The coverslips were removed and observed using laser scanning confocal microscope (Nikon Ti-E A1, Japan).

### 4.10. Statistical Analysis

The experiments were repeated three times separately and the results are presented as means ± standard deviations (SDs). The statistical significance of the results was evaluated using the independent t-test for the comparison of two samples and using a one-way ANOVA test for the comparison of more than two samples. A p-value less than 0.05 was considered statistically significant.

## Figures and Tables

**Table 1:** Primers used for the reverse transcription polymerase chain reaction.

Name	Primer Sequence
iNOS	F: 5′-CCTCCTCGTTCAGCTCACCT-3′
	R: 5′-CAATCCACAACTCGCTCCAA-3′
COX-2	F: 5′-CCTGGTGAACTACGACTGCTA-3′
	R: 5′-AGTGGAGAACGTCTTCAGATGAG-3′
TNF-α	F: 5′-ACTGGCAGAAGAGGCACTCC-3′
	R: 5′-GCCACAAGCAGGAATGAGAA-3′
IL-6	F: 5′-TCCATCCAGTTGCCTTCTTG-3′R: 5′-AAGCCTCCGACTTGTGAAGTG-3′
IL-1β	F: 5′-TTCAGGCAGGCAGTATCACTC-3′
	R: 5′-GAAGGTCCACGGGAAAGACAC-3′
β-Actin	F: 5′-GTGAAGGTGACAGCAGTCGGTT-3′
	R: 5′-GAAGTGGGGTGGCTTTTAGGA-3′

## References

[1] Dong L., Yin L., Zhang Y., Fu X., Lu J. (2017). Anti-inflammatory effects of ononin on lipopolysaccharide-stimulated RAW 264.7 cells. Mol. Immunol..

[2] Koh W., Shin J.S., Lee J., Lee I.H., Lee S.K., Ha I.H., Chung H.J. (2017). Anti-inflammatory effect of Cortex Eucommiae via modulation of the toll-like receptor 4 pathway in lipopolysaccharide-stimulated RAW 264.7 macrophages. J. Ethnopharmacol..

[3] Lawrence T., Willoughby D.A., Gilroy D.W. (2002). Anti-inflammatory lipid mediators and insights into the resolution of inflammation. Nat. Rev. Immunol..

[4] Mantovani A., Barajon I., Garlanda C. (2018). IL-1 and IL-1 regulatory pathways in cancer progression and therapy. Immunol. Rev..

[5] Rubio-Ruiz M.E., Peredo-Escarcega A.E., Cano-Martinez A., Guarner-Lans V. (2015). An Evolutionary Perspective of Nutrition and Inflammation as Mechanisms of Cardiovascular Disease. Int. J. Evolut. Biol..

[6] Marcason W. (2010). What Is the Anti-Inflammatory Diet?. J. Am. Diet. Assoc..

[7] Minihane A.M., Vinoy S., Russell W.R., Baka A., Roche H.M., Tuohy K.M., Teeling J.L., Blaak E.E., Fenech M., Vauzour D. (2015). Low-grade inflammation, diet composition and health: Current research evidence and its translation. Br. J. Nutr..

[8] Esser N., Paquot N., Scheen A.J. (2015). Anti-inflammatory agents to treat or prevent type 2 diabetes, metabolic syndrome and cardiovascular disease. Expert Opin. Investig. Drug.

[9] Sostres C., Gargallo C.J., Arroyo M.T., Lanas A. (2010). Adverse effects of non-steroidal anti-inflammatory drugs (NSAIDs, aspirin and coxibs) on upper gastrointestinal tract. Best Pract. Res. Clin. Gastroenterol..

[10] Mozaffarian D., Wu J.H.Y. (2018). Flavonoids, Dairy Foods, and Cardiovascular and Metabolic Health A Review of Emerging Biologic Pathways. Circ. Res..

[11] Lee Y., Gao Q.T., Kim E., Lee Y., Park S.J., Lee H.E., Jang D.S., Ryu J.H. (2015). Pretreatment with 5-hydroxymethyl-2-furaldehyde blocks scopolamine-induced learning deficit in contextual and spatial memory in male mice. Pharmacol. Biochem. Behav..

[12] Shapla U.M., Solayman M., Alam N., Khalil M.I., Gan S.H. (2018). 5-Hydroxymethylfurfural (HMF) levels in honey and other food products: Effects on bees and human health. Chem. Cent. J..

[13] Zirbes L., Nguyen B.K., de Graaf D.C., De Meulenaer B., Reybroeck W., Haubruge E., Saegerman C. (2013). Hydroxymethylfurfural: A possible emergent cause of honey bee mortality?. J. Agric. Food Chem..

[14] Kim H.K., Choi Y.W., Lee E.N., Park J.K., Kim S.G., Park D.J., Kim B.S., Lim Y.T., Yoon S. (2011). 5-Hydroxymethylfurfural from black garlic extract prevents TNFalpha-induced monocytic cell adhesion to HUVECs by suppression of vascular cell adhesion molecule-1 expression, reactive oxygen species generation and NF-kappaB activation. Phytother. Res. PTR.

[15] Ryu J.H., Kang D. (2017). Physicochemical Properties, Biological Activity, Health Benefits, and General Limitations of Aged Black Garlic: A Review. Molecules.

[16] Kim D.G., Kang M.J., Hong S.S., Choi Y.H., Shin J.H. (2017). Antiinflammatory Effects of Functionally Active Compounds Isolated from Aged Black Garlic. Phytother. Res..

[17] Kim J.H., Choo Y.Y., Tae N., Min B.S., Lee J.H. (2014). The anti-inflammatory effect of 3-deoxysappanchalcone is mediated by inducing heme oxygenase-1 via activating the AKT/mTOR pathway in murine macrophages. Int. Immunopharmacol..

[18] Abraham K., Gurtler R., Berg K., Heinemeyer G., Lampen A., Appel K.E. (2011). Toxicology and risk assessment of 5-Hydroxymethylfurfural in food. Mol. Nutr. Food Res..

[19] Michail K., Matzi V., Maier A., Herwig R., Greilberger J., Juan H., Kunert O., Wintersteiger R. (2007). Hydroxymethylfurfural: An enemy or a friendly xenobiotic? A bioanalytical approach. Anal. Bioanal. Chem..

[20] Li M.M., Wu L.Y., Zhao T., Wu K.W., Xiong L., Zhu L.L., Fan M. (2011). The protective role of 5-hydroxymethyl-2-furfural (5-HMF) against acute hypobaric hypoxia. Cell Stress Chaperones.

[21] Zhao L., Chen J., Su J., Li L., Hu S., Li B., Zhang X., Xu Z., Chen T. (2013). In vitro antioxidant and antiproliferative activities of 5-hydroxymethylfurfural. J. Agric. Food Chem..

[22] Li Y.X., Li Y., Qian Z.J., Kim M.M., Kim S.K. (2009). In Vitro Antioxidant Activity of 5-HMF Isolated from Marine Red Alga Laurencia undulata in Free Radical Mediated Oxidative Systems. J. Microbiol. Biotechnol..

[23] Li W., Qu X.N., Han Y., Zheng S.W., Wang J., Wang Y.P. (2015). Ameliorative effects of 5-hydroxymethyl-2-furfural (5-HMF) from Schisandra chinensis on alcoholic liver oxidative injury in mice. Int. J. Mol. Sci..

[24] Wolkart G., Schrammel A., Koyani C.N., Scherubel S., Zorn-Pauly K., Malle E., Pelzmann B., Andra M., Ortner A., Mayer B. (2017). Cardioprotective effects of 5-hydroxymethylfurfural mediated by inhibition of L-type Ca(2+) currents. Br. J. Pharmacol..

[25] Kinney J.W., Bemiller S.M., Murtishaw A.S., Leisgang A.M., Lamb B.T. (2018). Inflammation as a central mechanism in Alzheimer’s disease. Alzheimer’s Dement. Transl. Res. Clin. Interv..

[26] Abdulmalik O., Safo M.K., Chen Q., Yang J., Brugnara C., Ohene-Frempong K., Abraham D.J., Asakura T. (2005). 5-hydroxymethyl-2-furfural modifies intracellular sickle haemoglobin and inhibits sickling of red blood cells. Br. J. Haematol..

[27] Hannemann A., Cytlak U.M., Rees D.C., Tewari S., Gibson J.S. (2014). Effects of 5-hydroxymethyl-2-furfural on the volume and membrane permeability of red blood cells from patients with sickle cell disease. J. Physiol..

[28] Yamada P., Nemoto M., Shigemori H., Yokota S., Isoda H. (2011). Isolation of 5-(hydroxymethyl)furfural from Lycium chinense and its inhibitory effect on the chemical mediator release by basophilic cells. Planta Med..

[29] Kitts D.D., Chen X.M., Jing H. (2012). Demonstration of antioxidant and anti-inflammatory bioactivities from sugar-amino acid maillard reaction products. J. Agric. Food Chem..

[30] Stewart A.G., Beart P.M. (2016). Inflammation: Maladies, models, mechanisms and molecules. Br. J. Pharmacol..

[31] Brune B., Dehne N., Grossmann N., Jung M., Namgaladze D., Schmid T., von Knethen A., Weigert A. (2013). Redox control of inflammation in macrophages. Antioxid. Redox Signal..

[32] Miyake K. (2007). Innate immune sensing of pathogens and danger signals by cell surface Toll-like receptors. Semin. Immunol..

[33] Blaser H., Dostert C., Mak T.W., Brenner D. (2016). TNF and ROS Crosstalk in Inflammation. Trends Cell Biol..

[34] Varfolomeev E., Vucic D. (2018). Intracellular regulation of TNF activity in health and disease. Cytokine.

[35] Tanaka T., Narazaki M., Kishimoto T. (2014). IL-6 in inflammation, immunity, and disease. Cold Spring Harbor Perspect. Biol..

[36] Chien S.Y., Huang C.Y., Tsai C.H., Wang S.W., Lin Y.M., Tang C.H. (2016). Interleukin-1 beta induces fibroblast growth factor 2 expression and subsequently promotes endothelial progenitor cell angiogenesis in chondrocytes. Clin. Sci..

[37] Tsatsanis C., Androulidaki A., Dermitzaki E., Gravanis A., Margioris A.N. (2007). Corticotropin releasing factor receptor 1 (CRF1) and CRF2 agonists exert an anti-inflammatory effect during the early phase of inflammation suppressing LPS-induced TNF-alpha release from macrophages via induction of COX-2 and PGE(2). J. Cell. Physiol..

[38] Ishita I.J., Islam M.N., Kim Y.S., Choi R.J., Sohn H.S., Jung H.A., Choi J.S. (2016). Coumarins from Angelica decursiva inhibit lipopolysaccharide-induced nitrite oxide production in RAW 264.7 cells. Arch. Pharm. Res..

[39] Kim S.F., Huri D.A., Snyder S.H. (2005). Inducible nitric oxide synthase binds, S-nitrosylates, and activates cyclooxygenase-2. Science.

[40] Mittal M., Siddiqui M.R., Tran K., Reddy S.P., Malik A.B. (2014). Reactive oxygen species in inflammation and tissue injury. Antioxid. Redox Signal..

[41] Griffith B., Pendyala S., Hecker L., Lee P.J., Natarajan V., Thannickal V.J. (2009). NOX Enzymes and Pulmonary Disease. Antioxid. Redox Signal..

[42] Ulbricht R.J., Northup S.J., Thomas J.A. (1984). A review of 5-hydroxymethylfurfural (HMF) in parenteral solutions. Fundam. Appl. Toxicol..

[43] Jin M.S., Lee J.O. (2008). Structures of the toll-like receptor family and its ligand complexes. Immunity.

[44] Hu N., Zhang Y.M. (2017). TLR4 knockout attenuated high fat diet-induced cardiac dysfunction via NF-kappa B/JNK-dependent activation of autophagy. Biochim. Biophys. Acta Mol. Basis Dis..

[45] Sherman D.J., Xie R., Taylor R.J., George A.H., Okuda S., Foster P.J., Needleman D.J., Kahne D. (2018). Lipopolysaccharide is transported to the cell surface by a membrane-to-membrane protein bridge. Science.

[46] Wang L.L., Zhu R., Huang Z.Q., Li H.G., Zhu H.G. (2013). Lipopolysaccharide-Induced Toll-Like Receptor 4 Signaling in Cancer Cells Promotes Cell Survival and Proliferation in Hepatocellular Carcinoma. Dig. Dis. Sci..

[47] Jung W.K., Heo S.J., Jeon Y.J., Lee C.M., Park Y.M., Byun H.G., Choi Y.H., Park S.G., Choi I.W. (2009). Inhibitory Effects and Molecular Mechanism of Dieckol Isolated from Marine Brown Alga on COX-2 and iNOS in Microglial Cells. J. Agric. Food Chem..

[48] Chilton P.M., Embry C.A., Mitchell T.C. (2012). Effects of differences in lipid A structure on TLR4 pro-inflammatory signaling and inflammasome activation. Front. Immunol..

[49] Mao J., Liu J., Pang X., Li M., Song J., Han C., Wu D., Wang S. (2012). Nicotine induces the expression of C-reactive protein via MAPK-dependent signal pathway in U937 macrophages. Mol. Cells.

[50] Camacho-Barquero L., Villegas I., Sanchez-Calvo J.M., Talero E., Sanchez-Fidalgo S., Motilva V., de la Lastra C.A. (2007). Curcumin, a Curcuma longa constituent, acts on MAPK p38 pathway modulating COX-2 and NOS expression in chronic experimental colitis. Int. Immunopharmacol..

[51] Guha M., Mackman N. (2001). LPS induction of gene expression in human monocytes. Cell Signal..

[52] Aly S.M., Ahmed Y.A.G., Ghareeb A.A.A., Mohamed M.F. (2008). Studies on Bacillus subtilis and Lactobacillus acidophilus, as potential probiotics, on the immune response and resistance of Tilapia nilotica (Oreochromis niloticus) to challenge infections. Fish Shellfish Immunol..

[53] Shih R.H., Wang C.Y., Yang C.M. (2015). NF-kappaB Signaling Pathways in Neurological Inflammation: A Mini Review. Front. Mol. Neurosci..

[54] Potoyan D.A., Zheng W.H., Komives E.A., Wolynes P.G. (2016). Molecular stripping in the NF-kappa B/I kappa B/DNA genetic regulatory network. Proc. Natl. Acad. Sci. USA.

[55] Kim Y.S., Ahn C.B., Je J.Y. (2016). Anti-inflammatory action of high molecular weight Mytilus edulis hydrolysates fraction in LPS-induced RAW264.7 macrophage via NF-kappa B and MAPK pathways. Food Chem..

[56] Yamamoto Y., Gaynor R.B. (2001). Therapeutic potential of inhibition of the NF-kappaB pathway in the treatment of inflammation and cancer. J. Clin. Investig..

[57] Cao S., Zhang X., Edwards J.P., Mosser D.M. (2006). NF-kappaB1 (p50) homodimers differentially regulate pro- and anti-inflammatory cytokines in macrophages. J. Biol. Chem..

[58] Wang H.F., Zhang L., Xu S.C., Pan J., Zhang Q.X., Lu R.R. (2018). Surface-Layer Protein from Lactobacillus acidophilus NCFM Inhibits Lipopolysaccharide-Induced Inflammation through MAPK and NF-kappa B Signaling Pathways in RAW264.7 Cells. J. Agric. Food Chem..

[59] Hu Y., Lou J., Mao Y.Y., Lai T.W., Liu L.Y., Zhu C., Zhang C., Liu J., Li Y.Y., Zhang F. (2016). Activation of MTOR in pulmonary epithelium promotes LPS-induced acute lung injury. Autophagy.

[60] Li B., Xi P., Wang Z., Han X., Xu Y., Zhang Y., Miao J. (2018). PI3K/Akt/mTOR signaling pathway participates in Streptococcus uberis-induced inflammation in mammary epithelial cells in concert with the classical TLRs/NF-kB pathway. Vet. Microbiol..

[61] Luo L., Wall A.A., Yeo J.C., Condon N.D., Norwood S.J., Schoenwaelder S., Chen K.W., Jackson S., Jenkins B.J., Hartland E.L. (2014). Rab8a interacts directly with PI3K gamma to modulate TLR4-driven PI3K and mTOR signalling. Nat. Commun..

